# Rare disease genomics in an era of human pangenomics and telomere-to-telomere genome references

**DOI:** 10.1038/s41431-026-02125-7

**Published:** 2026-05-14

**Authors:** Chiara Folland, Gavin Monahan, James Breen, Mridul Johari, Hardip R. Patel, Gianina Ravenscroft

**Affiliations:** 1 Centre for Medical Research, University of Western Australia, Harry Perkins Institute of Medical Research, Perth, WA Australia; 2 Black Ochre Data Labs (Indigenous Genomics), The Kids Research Institute Australia, Adelaide, SA Australia; 3 National Centre for Indigenous Genomics, John Curtin School of Medical Research, Australian National University, Acton, ACT Australia

**Keywords:** Genetics research, Medical genomics

## Abstract

Despite considerable efforts investigating the genetic aetiology of rare diseases in the past decades, approximately 50% of cases remain without a genetic diagnosis. Many missing diagnoses can be attributed to the limitations of short-read sequencing (SRS), compounded by (mis)-alignment to incomplete and inaccurate reference genomes such as GRCh37/38. SRS cannot resolve many regions that are challenging to map, including large contiguous tandem repeats, segmental duplications (SDs), sites of complex structural variants (SV), or highly diverged population-specific loci. Long-read sequencing (LRS) technologies have delivered the first complete human genome assembly, T2T-CHM13. Compared to GRCh38, T2T-CHM13 resolves the remaining 8% of the genome, corrects structural errors and improves both SRS- and LRS-based read mapping and variant discovery. LRS has also facilitated the generation of high-quality, haplotype-resolved assemblies from globally diverse cohorts, enabling the construction of pangenome references for multiple ancestral groups. By representing more human genomic variation, a pangenome reference can improve mapping and variant calling accuracy. These new genome resources represent alternative reference paradigms that have the potential to uncover pathogenic variants underlying unsolved rare genetic diseases. Here, we examine the limitations of GRCh38 for rare disease variant discovery and explore how emerging resources like T2T-CHM13 and pangenomes can improve accuracy. We highlight key studies that have leveraged these references to improve diagnostic outcomes and discuss the potential for broader adoption. Finally, we consider the current barriers to research and clinical implementation and outline available resources and tools to expedite the transition to these new reference models.

## Introduction

Rare diseases (although individually rare) are cumulatively common, affect patients throughout life and may be severely disabling or life-threatening [1]. Patients with rare diseases frequently describe a prolonged ‘diagnostic odyssey’, taking in some instances decades for a definitive molecular diagnosis. The years anticipating a molecular diagnosis are typically characterised by uncertainty, multiple hospital visits, unnecessary and often invasive investigations, misdiagnoses and inappropriate management and treatments [2].

Improvements in technologies have been pivotal to increasing the genetic diagnosis of rare diseases. In 2003, genetics research was revolutionised by the completion of the Human Genome Project, a 15-year, international effort to sequence the human genome [3]. The official assembly released in 2003 by the International Human Genome Sequencing Consortium and subsequent updates were maintained by the National Center for Biotechnology Information (NCBI). Since 2009, the Genome Reference Consortium (GRC) has managed improvements to the sequence accuracy and completeness. The current GRC assembly, GRCh38, was released in 2013 and most recently patched in 2022 (GRCh38.p14). The GRCh38 reference has been used worldwide as a central component of genomic clinical service delivery and biomedical research.

High-throughput sequencing technologies gave rise to projects that set out to develop public resources of human genetic variation by sequencing healthy individuals. For example, the 1000 Genomes Project (1KGP) established a detailed public catalogue of human genetic variation that has been used extensively by the biomedical community to better understand the genetic underpinnings of both common and rare diseases [4]. Other sequencing consortia, including the Exome Sequencing Project (ESP) and the Trans-Omics for Precision Medicine Program (TOPMed), aimed to identify genetic variants associated with specific complex diseases, such as heart, lung and blood disease [5].

The utility of these large-scale sequencing projects has increased through the efforts of international consortia that have collated and standardised data from multiple projects and generated user-friendly visual browsers for exploring the variant datasets. Notably, the Genome Aggregation Database (gnomAD) has collated genome sequencing data from a variety of large-scale projects. Originally launched as the Exome Aggregation Consortium (ExAC), which contained exome sequencing data from ~60,000 individuals [6], gnomAD has undergone sequential iterations of data release. The most recent gnomAD version (v4.1) contains sequencing data from 807,162 individuals, including 76,156 genomes. In addition to expanding dataset size and quality, gnomAD has also updated its reference genome from GRCh37 to GRCh38, starting with version 3.0 (released in 2019). More recently, the All of Us Research Program was launched with the aim of sequencing genomes of over one million Americans from diverse ethnic backgrounds [7]. By providing variant frequency estimates, these reference datasets are useful to predict variant deleteriousness and are used in rare disease diagnostics to differentiate benign polymorphisms from pathogenic variants.

During the past decade, short-read sequencing (SRS), the GRCh37/38 assemblies and large population sequencing projects have been fundamental in the search for pathogenic variants underlying rare diseases. Despite their widespread uptake, the current diagnostic rate using SRS approaches remains at approximately 50%. The inherent limitations of SRS and the widespread use of these incomplete human reference genomes are likely a major contributor to the current diagnostic gap [8, 9].

Long-read sequencing (LRS) platforms, including single-molecule real-time sequencing from Pacific Biosciences (PacBio) and nanopore sequencing from Oxford Nanopore Technologies (ONT) or QitanTech, can resolve genomic regions that are recalcitrant to SRS analysis. LRS can generate sequencing reads >10 kbp spanning multiple polymorphisms, thus enabling accurate alignments. Combined with improved genome assembly methods [10], LRS can facilitate highly accurate haplotype-resolved assemblies. In 2022, the Telomere-to-Telomere (T2T) consortium released the first complete sequence of a human genome, T2T-CHM13 [11]. This achievement would not have been possible without LRS technologies that span tandem repeat copies of centromeres, ribosomal DNA and segmental duplications (SDs) [11].

The availability of high-quality assemblies has also contributed to the development of improved pangenome references. A pangenome is a graph-based data structure that represents a collection of genome sequences of multiple individuals that encompasses more of the genomic diversity of a species [12]. In 2023, the Human Pangenome Reference Consortium (HPRC), released the first draft of a human pangenome reference, which contains 47 highly accurate and near-complete diploid human genome assemblies from a cohort of genetically diverse individuals from the 1KGP [13].

This review will highlight, with exemplars, the following topics: (i) the current limitations of GRCh38 and how they impact genomic variant analysis and interpretation; (ii) improvements to genomic analyses enabled by new human genome assemblies and references, including the T2T-CHM13 assembly and human pangenomes; (iii) the prospective diagnostic utility of leveraging T2T-CHM13 and human pangenome references in rare disease research, including for underrepresented and indigenous populations; (iv) the key challenges associated with transitioning to these improved reference assemblies, including a lack of control variant call sets and genome annotation datasets, an absence of support for alternate references in established genomic informatics pipelines and the resource burden of undertaking large-scale re-alignment projects. This review will also summarise the key resources that may enable research and clinical laboratories to transition to T2T- or pangenome-based reference approaches.

## The status of the GRCh38 human reference genome

The GRCh38 human reference genome, released in 2013, consists of 25 sequences representing 22 autosomes, two sex chromosomes and a mitochondrial genome sequence (3.1 Gbp total length). In addition to the primary assembly, GRCh38 contains: (i) 42 sequences (7.0 Mbp) labelled with _*random* suffix that are linked to a chromosome but lack an exact chromosomal location; (ii) 127 sequences (4.5 Mbp) labelled with *chrUn* prefix that are identified as of human origin but without genomic location; and (iii) 261 sequences (109.5 Mbp) labelled with _*alt* suffix to represent alternate haplotype sequences for genetically divergent loci.

**Fig. 1:**
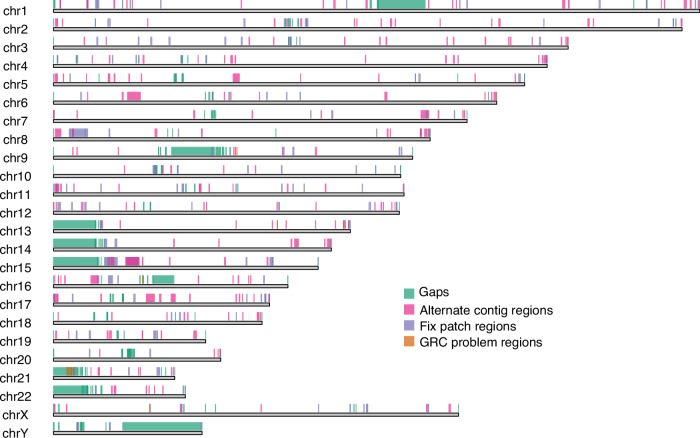
Distribution of Gaps and Problem Regions in GRCh38 Chromosomes. Regions of GRCh38 primary assembly chromosomes that are marked as gaps or GRC problem regions or overlap alternate contig or fix patches.

Since 2013, 254 patch sequences have been added to the reference. This includes 164 *fix patches* (65.9 Mbp total length) that introduced improvements to correct errors in the primary assembly, and 90 *novel patches* (23.7 Mbp total length) that provide alternate haplotype sequences for divergent loci. Collectively, these sequences contain 827 gaps (160.2 Mbp). To accommodate the inclusion of global ancestral diversity and missing sequences, the GRCh38 reference is often supplemented with 2,385 *decoy* sequences (5.8 Mbp) assembled from Simon Genome Diversity Project samples, HLA and KIR alternate haplotype sequences from the IMGT databases and Epstein-Barr Virus (*chrEBV*) sequence. Different combinations of sequences along with the primary assembly are used in genomic analysis, leading to the potential lack of reproducibility (Table 1). Moreover, non-primary sequences are not always used in genomic analyses, thereby excluding important biology and improvements added to the reference genome (Fig. 1).

**Table 1:** The composition of the GRCh38 primary assembly and other GRCh38 assembly versions used in genomic analysis.

Sequence type	Primary assembly	GRCh38.p14	full	no_alt_plus_hs38d1	no_alt	full_plus_hs38d1	GATK Resource Bundle	# of sequences	Total length (bp)	gapcounts	gaplen
Chromosomes (1-22, X, Y, M)	Yes	Yes	Yes	Yes	Yes	Yes	Yes	25	3,088,286,401	603	150,610,728
Unlocalized-scaffold (_random suffix)	Yes	Yes	Yes	Yes	Yes	Yes	Yes	42	6,978,808	38	335,137
Unplaced-scaffold (chrUn prefix)	Yes	Yes	Yes	Yes	Yes	Yes	Yes	127	4,485,509	54	157,106
Alternate contigs (_alt suffix)	No	Yes	Yes	No	No	Yes	Yes	261	109,535,387	124	8,847,328
EBV	No	No	Yes	Yes	Yes	Yes	Yes	1	171,823	–	–
Decoy (_decoy suffix)	No	No	No	Yes	No	Yes	Yes	2385	5,792,522	–	–
Fix patches	No	Yes	No	No	No	No	No	164	65,946,498	8	298,015
Novel patches	No	Yes	No	No	No	No	No	90	23,679,459	–	–
HLA sequences	No	No	No	No	No	No	Yes	525	2,096,467	–	–

GRCh38 also contains 59 Mb of computationally simulated sequences distributed throughout the genome, such as synthetic centromeric satellite arrays, ribosomal DNA sequences and recent SDs [11, 13, 14]. As a result, several regions of chromosomes 5, 14, 19, 21 and 22 are hard masked to Ns in analyses sets. Additionally, there are 94 nucleotides that are represented by non-ACGTN characters in the GRCh38 primary assembly. GRC has also identified 14 problematic regions (1.5 Mbp) containing contamination or false duplications. Overall, these gaps and errors in GRCh38 create an observational bias that limits genomic analysis within the boundaries of the reference [13, 15]. The issues with GRCh38 arise from the limitations of sequenced read lengths and the assembly technique: shotgun sequencing combined with bacterial artificial chromosome clone tiling using physical maps (see Supplementary Information).

After the initial assembly and sequencing of the human genome, high-throughput sequencing methods using short reads emerged. In this method, the genome is sequenced in 50–300 bp fragments. These SRS methods are inherently limited in their capacity to sequence, assemble and align reads to certain regions of the genome. These inaccessible regions are estimated to encompass 10–15% of the genome [14, 16] and arise primarily for two reasons: either the sequence itself is recalcitrant to sequencing due to properties like high GC content, or the region contains features (e.g. large contiguous tandem repeats or duplications) that challenge short-read bioinformatic analysis [8]. Short-read alignment algorithms are unable to confidently map sequences to their region of origin due to high sequence identity, length and structural diversity [8, 14].

## The completion of the human genome

The first complete sequence of a human genome was generated by sequencing the pseudo-haploid genome of a complete hydatidiform mole cell line (CHM13) [11]. To achieve a gapless assembly, the T2T consortium used multiple sequencing technologies, including PacBio’s HiFi and ONT’s ultralong sequencing, as well as chromosome conformation capture sequencing (Hi-C) and optical genome mapping (OGM) [11]. The resulting T2T-CHM13 assembly resolves the remaining 8% of the genome, corrects structural errors and adds almost 200 Mb of sequence compared to GRCh38 [11, 17]. The newly resolved sequence is predominantly heterochromatic, such as the pericentromeric and sub-telomeric regions, and consists of highly repetitive sequences, including duplicated gene families, ribosomal DNA and SDs [11, 18]. The T2T-CHM13 assembly introduces 1,956 new gene predictions, including 140 that are similar to known coding genes, some of which demonstrate functional importance and belong to gene families involved in disease (e.g. *WASHC1* and *GPRIN2*) [19].

Since the release of T2T-CHM13, there have been efforts to generate other assemblies with T2T or near-T2T status. Logsdon et al. used LRS to sequence 65 diverse human genomes and build 130 haplotype-resolved assemblies [20]. They reached T2T status for 39% of chromosomes and generated a catalogue of human variation, including inversions, deletions, mobile element insertions, SDs and copy number variations (CNVs), mapped to both GRCh38 and T2T-CHM13 [20]. These variant call sets provide a valuable genomic resource for studies leveraging T2T-CHM13 as a genomic reference.

### A telomere-to-telomere reference improves analysis of genomic variation, particularly complex SVs

The T2T consortium has demonstrated that use of the complete reference genome improves SRS- and LRS-based read mapping and genome-wide SNV, indel and SV discovery across globally diverse cohorts [15]. Their analysis uncovered over two million variants within genome regions that were previously left unresolved and improved variant calling accuracy across 622 medically relevant genes [15], 13% of a curated benchmark [9].

By capturing highly repetitive genome elements, T2T-CHM13 improves our capacity to identify regions of genomic instability and characterise both simple and highly complex structural variants (SVs). Simple SVs include deletions, insertions, duplications, inversions and translocations, whereas complex SVs comprise one or more simple SVs *in cis* that arise in a single event and involve three or more breakpoint junctions [21, 22]. Both simple and complex SVs are important contributors to rare genetic diseases [21–23]. The detection and accurate resolution of complex SVs is often complicated by (i) their size, often spanning several megabases of DNA; (ii) their association with repetitive genomic elements, including transposable elements, SDs and simple repeats; and (iii) the involvement of several breakpoint junctions, requiring a combination of genomic technologies to be accurately resolved [24–27].

Complex SVs typically reside in low-complexity regions (LCRs) that contain large repeats, tracts of sequence similarity or SDs, defined as inter- or intrachromosomal regions of homology that are over 1 kbp in length and have 90% sequence identity [14, 18, 21, 22]. These regions are susceptible to unequal meiotic crossover events between paralogous DNA segments via nonallelic homologous recombination (NAHR) that result in recurrent rearrangement [14, 28]. SDs are also associated with a high mutation rate, with a 50% higher SNV density compared to unique regions of the genome [18]. It is therefore important to use a reference genome with an accurate representation of these challenging genomic regions.

A comprehensive comparison of T2T-CHM13 and GRCh38 identified 81 Mbp of previously unresolved or structurally variable SDs, distributed throughout the genome [18]. By providing a more complete map of genome-wide SDs, T2T-CHM13 enables characterisation of complex SVs, including chromosomal heteromorphisms and other highly variable regions [18]. Although much of this variation is clinically benign, there is evidence that large-scale structural rearrangements are associated with reduced fertility and increasing rates of miscarriage [29], in addition to other genetic disorders [28, 30]. Therefore, T2T-CHM13 may facilitate the discovery of novel disease-causing SD-mediated chromosomal rearrangements.

## Beyond linear references: embracing human pangenome models

### Limitations of linear reference models

Conventional variant-calling workflows align reads to a single linear reference assembly, such as GRCh38 or T2T-CHM13. However, a linear genome assembly cannot represent the genetic diversity across human populations. For example, GRCh38 is a linear composite of genomic haplotypes assembled from >20 individuals, with a single individual contributing approximately 70% of the sequence [12]. It is estimated that up to 10% of the population-specific genome sequence is missing from the GRCh38 assembly [31]. Therefore, using GRCh38 as a reference for genome mapping and inference introduces pervasive reference allele bias, whereby alleles present in the reference genome are over-reported compared to alleles absent from the reference [32]. This bias impacts many primary genomic analyses, including read mapping, variant calling, genotyping and haplotype phasing, therefore reducing the accuracy of characterizing genomic variation [33]. This issue profoundly impacts SV detection because regions with frequently observed polymorphic SVs will vary in their sequence content between individuals and the linear reference [32, 33]. Over two-thirds of SVs have remained unidentified in SRS data because of the lack of representation of these alternate alleles in the GRCh37/38 assemblies [13].

Although T2T assemblies have unlocked access to some of the most intractable regions of the genome, they are still singular, monoploid references that cannot capture common genetic diversity across populations. Large cohort sequencing studies have demonstrated that a human individual may contain anywhere from 0.16 Mb to 14.2 Mb of novel sequence [31]. Therefore, even T2T-based approaches are limited in their capacity to characterise regions where the individual genetic sequence varies greatly from the reference, such as large indels, SVs and regions of high variability [34].

### The pangenome offers an alternative reference paradigm that can capture greater genomic diversity

A pangenome offers an alternate approach to storing and representing genomic information. A pangenome is an innovative graph-based data structure that can represent a collection of genome sequences of multiple individuals that encompasses more of the genomic diversity of a species [12]. Pangenome approaches enable comparison of a new genome to all those in a pangenome model [35]. This contrasts with traditional approaches, which align sequences to a linear consensus model of the genome.

Pangenomes are not a new concept, nor have they been uniquely applied to the study of human health and genetic variation. Pangenomes were initially described in the context of prokaryotes—particularly bacteria—in microbiology studies and have since been extended to eukaryotes across diverse fields such as ecology, phylogenomics and agriculture, as previously reviewed [31, 36].

Compared to a multiple sequence alignment, a pangenome graph condenses shared sequences as ‘nodes’ and represents relationships between nodes using ‘edges‘ [37]. Each haploid genome within the graph is represented by a path traversing nodes of the graph [37]. These graph-based pangenome representations may take many forms, including sequence graphs such as a *k*-mer-based de Bruijn graph, cyclic/acyclic variation graphs, or haplotype graphs, as previously reviewed [31, 35, 36]. Pangenome graph models and their accompanying algorithms and data structures have improved over time to better capture large and structurally complex genomes [31, 35, 36].

The same concept behind graph genomes has been applied to represent common genomic variation as alternate loci sequences that are supplementary to GRCh37/38 [31, 32]. Although this aims to mitigate reference allele bias, it is infeasible to capture the entirety of human allelic diversity using this alternative locus system because most sequence alignment programs were not designed to handle variant information provided in this format [31, 32, 38]. Embracing a pangenome reference model and the respective data structures, indices, algorithms and statistical methods, offers a more accurate way of capturing genomic diversity.

### Variant discovery and genotyping using pangenome graphs

Most existing pangenome-based variant calling tools are limited to genotyping known variants represented within the pangenome graph and are not designed for new variant discovery. For example, *Pangenie* is a short-read genotyper for SNVs, indels, and SVs represented in a pangenome graph that leverages an alignment-free approach [39]. Other short-read pangenome-based genotypers include *Graphtyper2* [40], *vg call* [38] and *Paragraph* [41]. Among these, *Graphtyper2* is unique for its ability to both genotype known variants and discover novel SNVs and indels by aligning short reads directly to the graph and dynamically updating the graph with newly identified variants [40]. More recently, a pangenome-aware *DeepVariant* model was developed, which leverages a pangenome graph to improve the accuracy of short read variant calling in samples mapped to a linear reference rather than using read-level information alone [42]. Tools for pangenome-based SV discovery remain limited, with current methods designed exclusively for LRS data [43, 44].

To ensure backward compatibility with existing genomics tools, graph-based read alignments can be ‘surjected’ onto a linear reference [13, 45, 46]. In this process, read mappings generated against a pangenome (typically in GAF format) are projected onto a linear reference, such as GRCh38 or T2T-CHM13, producing alignment files (BAM/CRAM) compatible with existing linear variant calling pipelines. This strategy reduces reference bias while improving variant detection accuracy compared to direct mapping against a singular linear reference [13].

### Improved genome analysis using pangenome graphs

Early human pangenome studies created variation-graph-based pangenome references using variant data from SRS population projects such as the 1KGP [32, 46, 47]. By representing more genetic variation, aligning SRS data to these variation graphs achieved higher mapping accuracy, particularly within challenging genomic regions, compared to contemporary linear reference-based pipelines [46, 47]. These graph-based approaches also demonstrated improved performance for both small variant calling and SV genotyping, with the latter benefiting the most from variation-aware mapping and graph-based genotyping [46].

More recent work has leveraged LRS for genome assembly and pangenome graph construction, alongside advancements in graph-based mapping, variant calling and genotyping algorithms, further enhancing performance [38, 45]. Others have explored the use of population-specific genome graphs, showing that as graph references become more tailored to the genetic background of the population, read alignment error rates decrease and variant calling sensitivity improves [48–51].

### A draft human pangenome

The HPRC was launched in 2019 with the mission of promoting a paradigm shift in genomics towards the use of a human pangenome [12]. In 2023, the HPRC released the first draft of a human pangenome reference [13]. The draft pangenome contains 47 highly accurate and near-complete diploid human genome assemblies from a cohort of genetically diverse individuals from the 1KGP [13]. Application of this draft pangenome to downstream SRS analysis workflows demonstrated improvements to both small variant and SV calling, compared to linear, GRCh38-based analysis [13]. The goal of the HPRC is to generate a stable release of a human pangenome reference containing genome sequences from 350 people by mid-2026. Accordingly, an intermediate release comprising >200 samples was made available in May 2025.

## The potential utility of T2T-CHM13 and pangenomes in rare disease research

**Fig. 2:**
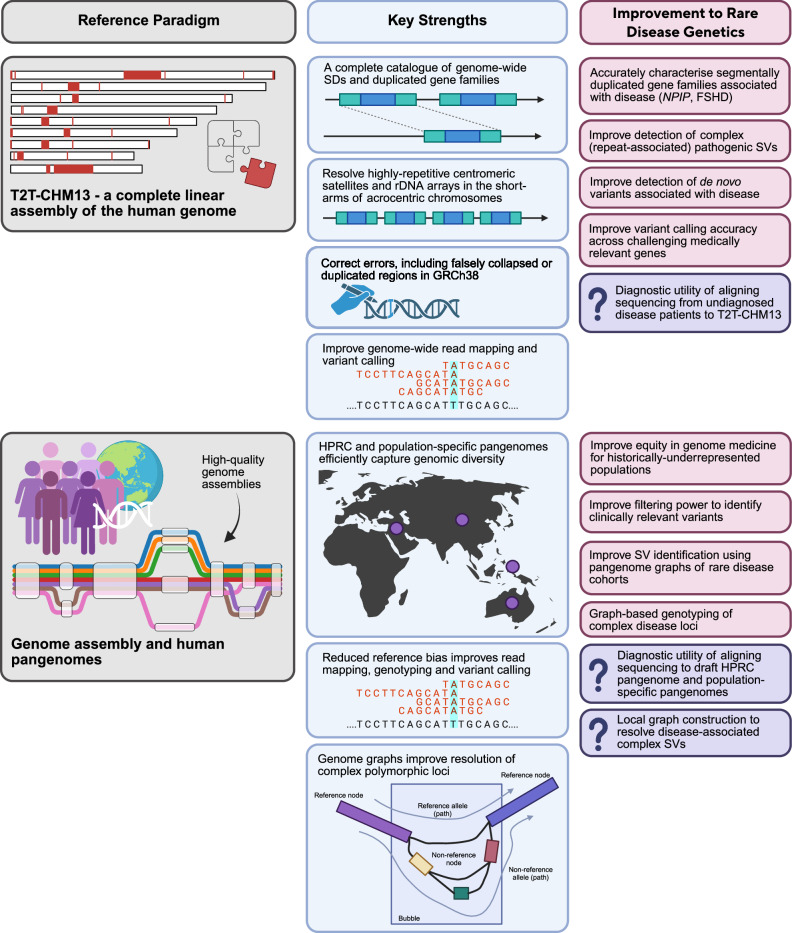
High-level overview of the two reference paradigms, T2T-CHM13 and pangenome graphs. The core advantages of using each reference in genetic analysis are highlighted in blue boxes. A summary of the key improvements offered by each paradigm to rare disease genetics and diagnostics is provided in the third column (red boxes). Future directions are designated in purple boxes with a question mark to denote a knowledge gap. Genome graphs adapted from Groza et al. [43] and Hickey et al. [37]. SD, segmental duplication; rDNA, ribosomal DNA; FSHD, facioscapulohumeral muscular dystrophy; SV, structural variant; HPRC, human pangenome reference consortium. Created in BioRender. Folland, C. (2026) https://BioRender.com/axo220r.

T2T-CHM13 and other high-quality genome assemblies, together with pangenome approaches, represent powerful new resources for advancing rare genetic disease research and diagnostics. Here, we summarise current applications of T2T-CHM13 and pangenomes in medical genetics, highlighting the case-specific strengths of each approach. Exemplar studies that have leveraged T2T-CHM13 and/or pangenomes to improve rare genetic disease analysis are summarised in Table 2. Fig. 2 provides a schematic comparison of the T2T‑CHM13 and pangenome graph reference paradigms, outlining their advantages for genetic analysis and summarizing key improvements, future directions and outstanding knowledge gaps in rare disease genetics.

**Table 2:** Published rare disease applications of T2T-CHM13 and/or pangenome references, highlighting diagnostic utility.

Authors	Date	Test Cohort	Disease(s)	Technology/Approach	Diagnostic Outcome	Utility of Novel Reference	Reference
*CHM13*							
Schuy et al.	2024	3 cytogenetically visible complex chromosome 21 rearrangements	Chromosome 21 developmental and malformation syndromes	LRS, SRS, OGM	No new diagnoses.	CHM13 contains the full acrocentric p-arm and centromere sequence of chromosome 21 (absent in GRCh37/38), enabling complete breakpoint resolution and mechanistic understanding of all three variants.	[52]
Saether et al.	2024	12 rare cytogenetically visible inversions	Various	SRS, LRS	No new diagnoses. Nine inversions were identifiable using SRS/LRS.	CHM13 resolves repetitive regions that are absent in GRCh37/38, enabling resolution of two inversions with breakpoints localised to repetitive regions.	[17]
Xia et al.	2024	2 cytogenetically visible structural rearrangements	Miscarriage, foetal malformation	LRS	No new diagnoses. Both SVs could be detected using LRS.	CHM13 resolves gaps in the centromeric sequence required to resolve breakpoints of a pericentromeric inversion.	[29]
Höps et al.	2025	145 known clinically significant variants	Various	LRS	No new diagnoses. 135 variants identifiable using LRS.	CHM13 was required to fully resolve an unbalanced translocation involving chromosomes 13 and Y.	[79]
Noyes et al.	2022	1 unsolved family (quad)	Autism	LRS, SRS, OGM	No single causative variant was identified. 195 de novo SNVs/indels were identified in the quad, a 35% increase from the previous analysis.	CHM13 increased the number of de novo variants identified by 5% and increased the true positive rate. Variants exclusive to CHM13 were in centromeres, SDs, and LCRs or recent repeats.	[16]
*Pangenome*							
Sui et al.	2026	51 unsolved families	Autism, Rhett Syndrome	LRS, de novo assembly, pangenome filtering	Three novel diagnoses (5.9%) and nine candidate variants.	Pangenome-based filtering greatly reduced the burden of variant filtering.	[64]
Groza et al.	2024	287 probands	Various paediatric rare diseases	LRS, SRS, de novo assembly, pangenome construction	One novel diagnosis (exonic rare SV in *KMT2E*).	Pangenome graph approach increases the number of SVs identified and improves precision/recall.	[43]
Jang et al.	2025	33 undiagnosed Korean families	Various rare diseases	LRS, de novo assembly, pangenome construction	Nine novel diagnoses (27.3%) were enabled via LRS.	Unclear contribution.	[63]

*LRS* long-read sequencing, *SRS* short-read sequencing, *OGM* optical genome mapping, *SNV* single-nucleotide variant, *SV* structural variant, *SD* segmental duplication, *LCR* low-complexity region.

## T2T-CHM13 resolves complex cytogenetically visible pathogenic SVs

Studies have leveraged T2T-CHM13 to resolve large structural rearrangements that were previously detected using cytogenetic testing [17, 29, 52]. Many of the disease-causing variants in these studies could not be resolved using GRCh37/38-based mapping because they overlapped regions missing from these outdated references [17, 29, 52]. Schuy et al. leveraged T2T-CHM13, LRS and OGM to resolve complex rearrangements on the p-arm of chromosome 21, associated with various developmental and malformation syndromes, including Down Syndrome [52]. All 21p rearrangements could not be resolved using GRCh38, due to incomplete coverage of the full acrocentric p-arm and centromere sequences [52]. Saether et al. used T2T-CHM13 and LRS to improve the resolution of nine rare pathogenic inversions, particularly those with breakpoints in repetitive regions [17]. Two inversions involved breakpoint regions that were missing from GRCh37/38 and therefore only detectable by aligning to T2T-CHM13, regardless of whether LRS or SRS was used [17]. Finally, Xia et al. used Nanopore LRS mapped to T2T-CHM13 to improve preimplantation genetic testing for chromosomal structural rearrangements in heterochromatic (pericentromeric and subtelomeric) regions underlying miscarriage and fetal malformation [29]. These SVs could not be resolved using the incomplete GRCh37/38 assemblies due to gaps in the sequence of centromeric and telomeric regions [29].

Crucially, none of the studies mentioned above used T2T-CHM13 to discover novel disease-causing variants; the pathogenic variants had already been identified using orthogonal, legacy methods. Nevertheless, these studies serve as an important proof-of-concept that T2T-CHM13 can enable sequence-based diagnostics to access previously intractable variants that historically required cytogenetic techniques.

### T2T-CHM13 and LRS assembly improve the detection of de novo variants

De novo variants are important genetic contributors to common and rare diseases [53, 54]. Porubsky et al. utilised LRS, SRS and Strand-seq to generate comprehensive genomic data for members of a four-generation family [55]. These data were leveraged to create a reference truth set of inherited and de novo variation by using a combination of (i) read- and assembly-based mapping and variant calling against T2T-CHM13 and (ii) pangenome graph-based variant detection [55].

Similarly, a combination of assembly- and read-based approaches was used to study de novo variation in an autism parent-child quad [16]. In this study, the use of T2T-CHM13 increased the total accessible genome and the rate of true positive variant calls compared to GRCh38 for both SRS and LRS data [16]. The combined use of LRS and T2T-CHM13 was integral to accessing more complex and repeat-rich regions of the genome.

### T2T-CHM13 and HPRC assemblies improve analysis of complex disease-associated loci

Since their release, T2T-CHM13 and other highly accurate human genome assemblies have been used to characterise complex loci associated with human disease that have previously been poorly understood [56, 57]. For example, the *NPIP* gene family consists of a core duplicon that is highly duplicated across chromosome 16. Duplications embedded in large SD blocks with high sequence identity mediate further microdeletions and microduplications associated with neurodevelopmental phenotypes [56]. Standard SRS approaches have been insufficient to characterise the structural diversity of *NPIP* haplotypes [56]. Dishuck et al. leveraged T2T-CHM13 and 80 previously assembled genomes to characterise 169 human *NPIP* structural haplotypes, identifying 4665 copies of *NPIP* and assigned them to one of 28 phylogenetic paralogs [56]. This detailed map of *NPIP* paralogs lays an important foundation for accurate characterisation and association of *NPIP* variation with human neurological phenotypes and disease.

T2T-CHM13 also improves analysis of the highly repetitive and segmentally duplicated region of chromosome 4q associated with facioscapulohumeral muscular dystrophy (FSHD) [11, 57]. FSHD is caused by contraction and hypomethylation of the D4Z4 macrosatellite array at chromosome 4q35, which enables the stable expression of the DUX4 transcription factor [57]. Current models of the FSHD locus used in diagnostic tests are based on GRCh38, which harbours the D4Z4 repeat array on two loci (4q and 10q) [57]. A complete catalog of D4Z4 macrosatellite repeats, constructed using T2T-CHM13 [57] and HPRC assemblies [58], reveals a 10-fold increase in genome-wide D4Z4 repeats across multiple chromosomes compared to GRCh38. Recently, Yeow et al. released *d4z4ling*, a tool for comprehensively predicting FSHD status using LRS aligned to T2T-CHM13 [59]. This raises important questions about the accuracy of current genetic tests and methylation profiles used to diagnose FSHD based on an incomplete model of the FSHD locus, which may lead to erroneous signals from paralogous loci [57, 58].

### Local pangenome construction and graph-based genotyping of complex polymorphic disease loci

Compared to linear references, pangenomes are uniquely suited to analysing complex, polymorphic loci, as they capture the diversity of possible haplotype arrangements [34, 60]. Genotyping tools leveraging pangenome graphs have been developed for these complex genomic loci, including hundreds of medically relevant genes [61, 62]. For example, Locityper extracts a panel of haplotypes from a pangenome for a specific polymorphic locus to align sample reads (SRS or LRS) to those reference haplotypes and estimate the likelihood of the locus genotype [61]. The assigned genotype has the highest joint likelihood and the most probable read alignments, which can then be used for visual analysis and/or variant-calling [61].

Local pangenome graphs of complex polymorphic loci instead of the whole genome graphs, as demonstrated for the amylase locus [62], can also improve genotyping of complex SVs. Extending this strategy to medically relevant, polymorphic genes, including those implicated in rare genetic diseases, could further enhance graph-based genotyping and read assignments to improve variant calling.

### Pangenome graphs for identifying clinically relevant variants in rare disease patients

Pangenome graphs integrating genome assemblies from rare disease patients have been used to identify pathogenic variants missed by standard reference approaches. Groza et al. built a pangenome graph consisting of 574 haploid assemblies from rare disease patients augmented with the HPRC assemblies (*n* = 94) to identify rare and putatively functionally-relevant SVs [43]. Compared to approaches leveraging linear references, they were able to achieve a higher level of reproducibility, reduce error rates and increase the number of SVs identified, including common SVs and putatively pathogenic variants [43]. They also demonstrate that combining graph and reference-based approaches improves the precision of rare SV calling [43]. However, the identification of only a single novel diagnostic SV in *KMT2E* suggests a limited diagnostic yield for this approach.

In the preprint by Jang et al., a similar approach was applied to 40 individuals from 33 previously undiagnosed Korean families, identifying clinically actionable variants in nine families (27.3%) that had remained unsolved after SRS [63]. Although this study reports the highest diagnostic yield to date among rare disease studies using pangenome and/or T2T-CHM13–based approaches, the provided discussion does not clearly articulate the incremental utility of the graph-based strategy beyond the inherent advantages of LRS-based variant detection. Because the added value of the pangenome approach is not clearly disentangled from the effect of LRS, the specific contribution of the pangenome strategy remains challenging to assess.

In other studies, the utility of pangenomes is demonstrated by the reduction of the burden in variant filtering. Sui et al. generated LRS data and de novo assemblies of 51 families with unsolved autism or Rhett syndrome [64]. By leveraging 108 HPRC and HGSVC assemblies as controls, they were able to dramatically reduce the burden of SV variant filtering ( ~ 97% of common SVs were filtered per proband), simplifying the identification of clinically relevant SVs [64]. Using the phased assemblies, they were able to identify causative pathogenic variants in three families (5.9%) previously missed by SRS [64].

### Recommendations and future work

Beyond the studies described above, there has been limited uptake of the complete T2T-CHM13 reference genome in rare disease genetics research since its release in 2022. However, the improved resolution of complex SVs, particularly within highly repetitive regions and sequences absent from GRCh37/38, suggests that T2T-CHM13-based analyses may uncover disease-causing variants in patients who remain undiagnosed after standard GRCh37/38-based approaches. Systematic evaluation in cohorts of previously unsolved rare disease cases will be necessary to determine the diagnostic gain of adopting T2T-CHM13 as a reference.

It is imperative to assess the diagnostic utility of pangenome graph approaches, especially for resolving complex, polymorphic disease loci, which may reveal distinct diagnostic advantages. Furthermore, the diagnostic utility of re-aligning existing sequencing data from undiagnosed rare disease patients to pangenome references has not yet been systematically assessed. However, the tools required to undertake such studies are already available. Short-read pangenome mappers, such as *vg giraffe*, along with tools like *vg surject* for projecting graph-based alignments onto linear reference coordinates, enable seamless integration with existing downstream variant-calling and annotation workflows. This provides a practical foundation for evaluating the potential of pangenome references to improve diagnostic outcomes in rare disease genomics.

## Overcoming barriers to adoption

### Barriers to adoption of T2T-CHM13

Delayed adoption of T2T-CHM13 is not unexpected given the historically slow transition from GRCh37 to GRCh38. Many research and diagnostic laboratories continue to rely on GRCh37, despite GRCh38 being available for over a decade. There are many obstacles preventing the transition to newer references. Importantly, re-alignment projects are computationally intensive, incurring considerable financial and time burdens, which often outweigh the perceived benefits of transitioning to the improved reference [65]. These resource burdens are especially pronounced for large consortia sequencing projects, such as gnomAD, where updating would involve reprocessing hundreds of thousands of genomes. For example, sequence data aligned to GRCh38 was only made available in gnomAD v3.0, released in late 2019, six years after the GRCh38 assembly was released. Similarly, realignment of 1KGP reads to GRCh38 was published in 2017, four years after its release. Other major collaborative efforts in rare disease genomics, such as Solve-RD, have also been slow to adopt GRCh38, suggesting that the transition to T2T-CHM13 will likely follow a similarly gradual trajectory.

The full diagnostic potential of T2T-CHM13 will only be realised if large consortia remap their existing datasets to this assembly. As custodians of valuable genomic resources, these consortia carry a social responsibility to adopt and promote updated reference genomes, ensuring their data remains relevant and maximally useful. Despite the upfront costs, the long-term benefits of such large-scale remapping efforts, including advancing human genomic research and enabling precision medicine, make them a worthwhile investment.

Although the T2T consortium performed read alignments and short variant calls for 3202 genomes from the 1KGP, there remains a lack of T2T-aligned population data. This reduces filtering power in clinical variant analysis and thus creates a barrier to widespread uptake of T2T-CHM13 in rare disease research. One option is to use lift-over tools to convert variant co-ordinates to GRCh38 [66–68] and then compare allele frequencies with available datasets. However, the accuracy of variants lifted from T2T-CHM13 to GRCh38 is low compared to reanalysing the sequences using standard alignment and variant calling approaches [66]. Issues with lifting variants arise because there are considerable allelic differences between the two references [66]. Also, the region may be fully or partially missing from the incomplete reference [69]. An alternative to variant lift-over is lifting over alignments from T2T-CHM13 to GRCh38, using *LevioSAM2* [66]. Whilst benefitting from the use of an annotation-rich GRCh38 reference, *LevioSAM2* lift-over preserves many of the quality improvements of T2T-CHM13, resulting in improved variant calling accuracy for both small variants and SVs compared to direct-to-GRCh38 mapping [66].

Another considerable obstacle to new reference adoption in rare disease research is that many bioinformatic pipelines, analysis platforms and tools do not support T2T-CHM13 and instead rely on GRCh37/38. For example, the genomic tools maintained by the Broad Institute, including *seqr* [70] and *gatk-sv* [71], do not currently support T2T-CHM13. These tools are commonly used for rare disease variant detection and analysis, data sharing, disease-gene match-making, and other collaborative efforts and therefore their support for T2T-CHM13 is imperative for the adoption of this improved assembly within the rare disease research community. Other genomic analysis tools used in rare disease research that do not currently actively support T2T-CHM13, include alignment tools, variant analysis platforms, variant annotation tools and in silico predictors and are summarised in Supplementary Table 1.

### Barriers to the adoption of pangenome approaches

The availability of the HPRC draft human pangenome reference provides a valuable foundation for adopting pangenome-based approaches in the genetic analysis of undiagnosed rare disease patients. However, the current draft captures allelic diversity from 232 individuals and does not encompass the full spectrum of genetic variation across global populations. The stable future release aims to expand this to 350 individual genomes. Achieving comprehensive representation of global genetic diversity will require the development of additional population-specific pangenome references. Constructing these representative references will be a substantial undertaking, involving: (i) careful selection of individuals from diverse ancestral backgrounds to ensure broad representation of genetic diversity; (ii) strict attention to ethical, legal and social considerations for data acquisition, community engagement and priorities, informed consent, and data sharing; (iii) extensive data generation using multiple long-read and genomic technologies to achieve error-free phased assemblies; and (iv) advanced bioinformatics expertise for genome assembly, pangenome graph construction, and comprehensive downstream analysis. Until population-specific pangenomes become available, the full potential of pangenome-based approaches for variant discovery and interpretation will remain unrealised.

Ideally, a human pangenome reference should be based on a diverse set of complete, error-free human genome assemblies that can represent most of the allelic diversity. However, genome assembly is complicated by limitations of sequencing technologies and bioinformatics methods leading to phasing inaccuracies and the inability to resolve large SDs, satellite arrays and ribosomal DNA regions [11, 13, 20]. Therefore, achieving T2T status is resource-intensive and only a few assemblies of high quality exist [20].

Fully embracing pangenome references requires a paradigm shift in genomic analysis. The conceptual frameworks and analytic methods for key steps such as read mapping, indexing, variant calling, handling of recombination and data visualisation differ from those used with traditional linear reference genomes and remain areas of active development. Successful implementation of pangenome-based approaches will therefore depend on upskilling and training within the genomics community to ensure proper use of new graph-based alignment, visualisation and variant calling workflows, as well as accurate interpretation of downstream results.

### Resources and recommendations for adoption

To assist research and clinical teams planning T2T‑CHM13 realignment or adopting pangenome‑based genomic analysis, we provide a curated set of resources in Table 3, including T2T‑aligned datasets, lift‑over tools, variant‑analysis platforms and pipelines with active T2T‑CHM13 support, alongside key HPRC pangenome releases and graph‑based toolkits and workflows. We further recommend a minimal validation framework that includes assessing robust read‑mapping and variant‑calling performance on a small internal test set, confirming concordance for known pathogenic variants and ensuring consistent downstream annotation and reporting when using T2T‑aligned or graph‑derived coordinates.

**Table 3:** Resources for applying improved genome reference assemblies and pangenomic approaches to rare disease genetic research.

Resource	Description	Source
Data Repositories and Resources		
T2T-CHM13-based genomic resources	A data repository containing T2T-CHM13-based genomic resources generated from sequencing 65 diverse human genomes from the Human Genome Structural Variation Consortium (HGSVC) and generating haplotype-resolved genome assemblies. A pangenome graph containing all 65 samples, as well as 42 HPRC samples, was constructed using Minigraph-Cactus. Resources include simple and complex variant calls, genome graphs, genotyping results and annotations.	HGSVC3 Data Release
T2T-CHM13-based genomic resources	A GitHub repository containing links to various T2T-CHM13-based genomic resources generated by the T2T consortium project. This includes links to the latest T2T-CHM13 (v2) assembly, genome annotations, variant calls and lift-over resources.	GitHub
GA4K Pangenome Resources	A data repository containing genome graphs and variant call sets from individuals enrolled in a rare disease program (Genomic Answers for Kids, GA4K).	Zenodo
HPRC Assemblies	A GitHub repository containing data produced by the HPRC in their first release, including assemblies for 47 samples and their second release, including 234 samples.	Release 1 Release 2
HPRC Data Explorer	A data explorer to find and select HPRC sequencing data, assemblies, annotations and alignments	Webpage
HPRC Pangenome Resources	A GitHub repository containing data produced by the HPRC in their first release, including genome graphs generated using three different approaches.	GitHub
VEP T2T-CHM13 Annotation	A custom VEP configuration from the Ensembl rapid release compatible with T2T-CHM13 (Homo_sapiens_GCA_009914755.4).	Ensembl
AnnoVar T2T-CHM13 Annotation	A dataset for annotation of T2T-CHM13-aligned variants using AnnoVar.	Website
UCSC T2T-CHM13 track hub	A collection of T2T-CHM13-based genomic resources and tools, including lift-overs, annotations and BLAT.	Website
GIAB T2T-CHM13 Resources	A collection of Genome in a Bottle (GIAB) stratifications for T2T-CHM13, including mappability, complexity, repeats and functional regions.	Website
Pipelines & Workflows		
DRAGEN Pipeline	A genome sequencing data processing workflow that leverages a multi-genome mapping with pangenome references and machine learning-based variant detection.	Website
Minigraph-Cactus Pangenome Pipeline	A pipeline that uses *minigraph* to construct a pangenome graph of SVs in a set of input assemblies, then maps the assemblies back to the graph and finally uses Cactus to construct a new graph containing variants of all sizes. The HPRC has tested both SRS- and LRS- based mapping using a graph generated using this Minigraph-Cactus pipeline.	GitHub
vg snakemake pipeline	A Snakemake workflow for the *vg toolkit*, used to (i) index a pangenome using *vg*, (ii) map SRS to a pangenome using *vg giraffe*, (iii) call small variants (SNPs/indels) using *DeepVariant*, (iv) genotype SVs with *vg call* and (v) call SVs with *manta*.	GitHub
toil-vg	A Toil-based distributed and cloud computing framework for running common *vg* workflows.	GitHub
Genome Graphs Structural Analysis	A Snakemake workflow for constructing genome graphs and analysing their structure in depth.	GitHub
Genome Graph Variant Calling	A Snakemake workflow for mapping reads to genome graphs, surjecting graph alignments to linear formats and subsequent variant calling.	GitHub
Bionano Solve	Data processing pipeline for Bionano optical genome mapping data, including mapping, de novo assembly and SV calling.	Website
Tools & Platforms		
VARista Platform	Variant analysis and prioritisation platform that supports T2T-CHM13 aligned data.	Website
GeniePool 2.0	Integrates T2T-CHM13 with gnomAD v4, Sequence Read Archive (SRA), *AlphaMissense* and enables variant co-occurrence queries.	Website
VarSeq	NGS processing workflows and variant prioritisation platform.	Website
levioSAM2	Alignment lift-over tool. Enables alignment to T2T-CHM13 and lift-over to older references (e.g. GRCh38), improving the accuracy of subsequent variant calling. This enables access to a rich set of annotations, key for variant interpretation, which are built on older references and are underdeveloped for newer references.	GitHub
FixItFelix	A remapping approach that leverages a modified version of GRCh38 that fixes erroneous regions of the reference affected by falsely collapsed and falsely duplicated events. The approach improves mapping and variant calling in GRCh38-based genetic analysis.	GitHub
vg toolkit	A toolkit for working with variation graphs—bidirected DNA sequence graphs that compactly represent genetic variation across a population. The toolkit can be used to create, manipulate and use variation graphs as references, including indexing, mapping and variant calling.	GitHub
Pangenie	A short-read genotyper for small variants (SNPs/indels) and SVs represented in a pangenome graph.	GitHub
gaftools	A toolkit for processing pangenome alignments, including functions for indexing, sorting, realignment, viewing and statistical analysis.	GitHub
gfatools	A toolkit for manipulating sequence graphs in the GFA or rGFA format, including parsing, subgraph and conversion to FASTA/BED.	GitHub
SVarp	A tool for discovering haplotype-resolved SVs on top of a pangenome reference using long read sequencing.	GitHub
Panacus	A tool for computing statistics for GFA-formatted pangenome graphs.	GitHub
Odgi	A toolkit for analysing and manipulating large pangenome graphs.	GitHub
GraphTyper	A graph-based variant caller capable of discovering and genotyping population-scale SRS datasets.	GitHub
Paragraph	A graph-based SV genotyper for SRS data.	GitHub
Locityper	A graph-based genotyper for complex polymorphic genes.	GitHub
SequenceTubeMap	A visualisation tool that generates a ‘tube map’-like depiction of sequence graphs which have been created with *vg*.	GitHub
Bionano Access	Tertiary analysis software for Bionano optical genome mapping data.	Website
Bionano VIA	Tertiary analysis software for Bionano optical genome mapping, NGS and microarray data.	Website

*HPRC* Human Pangenome Reference Consortium, *VEP* Variant Effect Predictor, *UCSC* University of California, Santa Cruz, *SRS* short-read sequencing, *LRS* long-read sequencing, *SV* structural variant.

## Improved equity in genomic medicine

### Current genomic health inequities facing Indigenous communities

Despite growing efforts toward global representation, current genomic variation databases remain heavily skewed toward individuals of Northern European ancestry, limiting their utility for diverse populations [72]. Because there are clear differences in genetic variation and their frequencies across populations [73], increasing sample diversity is integral for improving representation of global human variation [72]. Increasing the diversity of genomic datasets improves the performance of several intolerance metrics [72]. By discerning genome regions that are intolerant to variation, these metrics are important for the interpretation of putative disease-causing genetic variants [74]. Therefore, increasing ancestral diversity in population variation databases will improve the equity and effectiveness of genomic diagnostics across populations.

For example, Indigenous Australian genomes have the highest proportion of population- and continent- private genetic variation observed outside of Africa [75]. Despite their rich and unique genomic diversity, Aboriginal and Torres Strait Islander communities are historically underrepresented in genomics research [69, 75]. Current population genome databases, including the 1KGP, gnomAD and the draft HPRC pangenome, do not capture unique variation from Indigenous Australian populations [69, 75]. Thirty-four percent of SNVs observed across 159 Indigenous Australians from four distinct communities were not present in the 1KGP or the Human Genome Diversity Project [75]. It is important to have diverse representation in control databases because allele frequency is used as an indicator for pathogenicity during variant filtering and prioritisation for clinical diagnostic investigations. The systemic and systematic lack of representation of Indigenous genomic data stems from the lack of appropriate engagement [76]. This gap is enforced through the unnecessary requirement of open-access data without consideration of Indigenous data sovereignty. By implementing principles of Indigenous data sovereignty models in the rare disease and precision health community, healthcare inequity can be reduced for the benefit of all. Addressing this gap is critical to ensuring equitable access to precision health medicine and improving genomic health outcomes in Indigenous and non-Indigenous communities.

### T2T-CHM13 and pangenomes may enhance equity in genome medicine by improving genetic analysis of Indigenous genomes

LRS and T2T-CHM13 offer novel avenues for characterising Indigenous genomic diversity. Reis et al. applied whole-genome ONT sequencing and used the T2T-CHM13 reference to characterise SVs across four Aboriginal communities [69]. The use of T2T-CHM13 significantly enhanced mappability and SV detection by contributing an additional 125 Mb of sequence accessible for analysis with ONT data [69]. The SV catalogue derived from the Indigenous communities was enriched for novel SVs—with an upper bound novelty estimate of 62%—highlighting substantial variation not previously captured in major cohort databases [69].

The study revealed that the genetic landscape of Indigenous Australians is not only distinct from non-Indigenous populations but also exhibits marked differences between individual Indigenous communities [69]. On average, approximately 185 ( ±  31) of the 311 uniquely Indigenous SVs identified per individual were private to their community [69]. Among the 121 Indigenous samples, the analysis detected 69 coding SVs affecting genes under loss-of-function constraint, suggesting potential clinical relevance [69]. These findings underscore that much of the SVs within Indigenous populations remain unsampled and emphasise the need for broader inclusion of Indigenous genomes in genomic research.

The LRS data generated as part of this study provided a unique opportunity to characterise short tandem repeat (STR) allelic diversity across and within Indigenous Australian populations. They found that STR allelic composition varied considerably between the Indigenous and non-Indigenous samples as well as between the Indigenous communities themselves [69]. Notably, of the 231 STR sites that showed inter-community differences in allelic composition, 67% sites demonstrated greater diversity in Indigenous than non-Indigenous Australians [69]. Many STRs demonstrated local variability, such as an increased allelic diversity unique to a single community, or expansions that were unique to a single individual or a small group of individuals [69]. These differences highlight the need for improved genomic tools and population-specific references to accurately detect and interpret STR expansions or contractions and other complex variant types within underrepresented groups.

Reference bias is particularly pronounced when analysing genome sequencing data from individuals whose allelic diversity is underrepresented or absent in current reference genomes. Since the release of the HPRC draft pangenome, several population-specific pangenomes have emerged, including Chinese (CPC) [51], Arab (APR) [50], Emirati [77] and Pacific Islander [49] populations. These population-specific pangenomes capture novel non-reference sequences and genetic variation that are absent in the HPRC draft pangenome, T2T-CHM13, and/or GRCh37/38 (see Table 4), offering more appropriate frameworks for variant analysis (and increasing the number of variants identified) in individuals from similar ethnic backgrounds, particularly in the context of rare disease and precision medicine [60, 78]. For example, there is a high prevalence of Mendelian recessive genes amongst uniquely duplicated genes in the APR (15.1%), compared with the HPRC (14.6%) and CPC (11.3%), which increases the risk of manifesting rare diseases [50, 60]. By capturing this unique genetic background, the APR offers improved estimation of rare disease burden and risk [60].

**Table 4:** Novel sequence and variants added by population-specific pangenomes.

Pangenome	Cohort	Reference(s)	Non-reference Sequence (Mbp)	Novel variants relative to HPRC		Novel gene duplications relative to HPRC	Reference
				**SNVs/Indels (Mbp)**	**SVs**		
Arab (APR)	53	GRCh38, CHM13	212.9	8.94^a^	235,195^a^	1135	[50]
Pacific	23	GRCh38, CHM13	92.5	3.44	4721	326	[49]
Chinese (CPC)	116	GRCh38, CHM13	194.7	5.9	34,223	1079	[51]
Emirati	58	GRCh38, CHM13	222.7				[77]
HPRC	47	GRCh38	175^b^			1115^c^	[13]

^a^Not previously within CHM13 and GRCh38 reference genomes or the HPRC and CPC pangenomes and other databases including the Database of Genomic Variants (DGV) and 1KGP.

^b^Only euchromatic autosomal sequence included (higher confidence assembly and alignment).

^c^Relative to GRCh38.

With the generation of new, high-quality genome assemblies from Aboriginal and Torres Strait Islander populations, the development of a draft Indigenous Australian pangenome is now within reach. Such a reference would be well suited to capturing the substantial inter- and intra-community genetic diversity observed across these populations, eliminating the need for multiple community-specific linear references. This would both simplify and enhance variant discovery and interpretation for these historically underrepresented communities, with the potential to reshape the landscape of genetic diagnosis, research and precision medicine in Australia and beyond.

## Conclusion

Genome reference assemblies form the bedrock of genomic analyses; using a gapless and representative reference is essential for ensuring accurate read mapping and variant calling. Despite the release of a complete reference, T2T-CHM13 and draft human pangenomes, GRCh37/38 are still widely used amongst the genomics community.

In this review, we have highlighted the major drawbacks of continued use of GRCh37/38, including the presence of unplaced/unlocalised and computationally simulated sequence, sequence gaps and errors and inconsistencies in the sequence between analysis sets. We explained how leveraging T2T-CHM13 improves genome-wide read mapping and SNV/indel and SV detection, particularly in regions of complex SVs and summarised studies that have leveraged this to improve rare disease genetic diagnoses.

We also discussed the drawbacks of relying on a linear consensus model of the human genome, which is inherently unsuited to capturing global sequence diversity, resulting in biases in variant calling and poorer outcomes for populations with divergent haplotypes. We discussed how pangenomes may be used as an alternate reference model and summarised the existing evidence for how pangenome-based analyses may outperform linear-based approaches in specific use cases such as highly polymorphic disease-associated loci.

Despite the promise of novel and innovative approaches, there are few studies evaluating their diagnostic utility in rare disease patient cohorts, highlighting an important arena for future research. We encourage all readers to consider adopting newer reference models and provide a list of resources (Table 3) to assist with this transition.

## Supplementary information

Supplementary Data
