# Supplementary material for: Rare disease genomics in an era of human pangenomics and telomere-to-telomere genome references
Source: Eur J Hum Genet. 2026 May 14;34(8):1033–46. doi: 10.1038/s41431-026-02125-7 (PMC13424327; doi:10.1038/s41431-026-02125-7)
Supplement: Supplementary file 1 — Supplementary Data [file 41431_2026_2125_MOESM1_ESM.docx]

**Supplementary Table 1.** Genomic analysis tools used in rare disease research that do not actively support T2T-CHM13

| Resource | Source |
| --- | --- |
| Variant Calling and Alignment Tools |  |
| Dragmap | [GitHub](https://github.com/Illumina/DRAGMAP) |
| gatk-sv | [GitHub](https://broadinstitute.github.io/gatk-sv/),) |
| Variant Analysis Platforms |  |
| Seqr | [GitHub](https://github.com/broadinstitute/seqr) |
| Scout | [GitHub](https://github.com/Clinical-Genomics/scout) |
| Open CRAVAT | [GitHub](https://www.opencravat.org/) |
| IVA | [GitHub](https://github.com/opencb/iva) |
| GENESIS | [Website](https://www.tgp-foundation.org/g-e-n-e-s-i-s) |
| illumina Emedgene | [Website](https://help.emg.illumina.com/emedgene-analyze-manual/tertiary-analysis-pipeline/which_reference_genomes_can_i_use) |
| Franklin by Qiagen | [Website](https://franklin.genoox.com/) |
| Mosaic | [Website](frameshift.io) |
| Varsome | [Website](http://varsome.com/) |
| Collaborative Tools |  |
| GeneMatcher | [Website](https://genematcher.org/) |
| In Silico Predictor Tools |  |
| CADD | [Website](https://cadd.gs.washington.edu/) |
| AlphaMissense | [Website](https://alphamissense.hegelab.org/) |
| REVEL | [Website](https://sites.google.com/site/revelgenomics/) |
| VEST |  |
| MPC |  |
| PrimateAI | [GitHub](https://github.com/Illumina/PrimateAI) |
| Eigen | [Website](http://www.columbia.edu/~ii2135/eigen.html) |
| PolyPhen2 | [Website](http://genetics.bwh.harvard.edu/pph2/) |
| SIFT | [Website](https://sift.bii.a-star.edu.sg/) |
| FATHMM | [Website](https://fathmm.biocompute.org.uk/) |
| ProtVar | [Website](https://www.ebi.ac.uk/ProtVar/) |

**GRCh38 Assembly**

GRCh38 was assembled by first fragmenting the genome into ~100 kbp sequences that were cloned into bacterial cells, known as bacterial artificial chromosomes (BACs). Individual BAC clones were then further sheared and sequenced using the Sanger method, generating reads up to 1,000 bp and assembled to produce BAC clone contigs/scaffolds. Utilising physical and genetic linkage maps where possible, BAC clone scaffolds were tiled to produce the genome assembly. However, this approach was constrained by the uneven distribution of genetic markers throughout the genome, resulting in unplaced or unlocalised contigs, assembly gaps, and synthetic sequences where contiguous assembly was not possible.
